# Establishment of an MR-Conditional Porcine Model for Real-Time Assessment of Cerebral Blood Flow During Extracorporeal Circulation

**DOI:** 10.3390/jcdd13050182

**Published:** 2026-04-27

**Authors:** Michael Hofmann, Martin O. Schmiady, Dominik T. Schulte, Tobias Aigner, Rima Bektas, Manuela Wieser, Martina Lentini, Francesca Del Chicca, Christoph Loeschmann, Michael Hübler, Ruth O’Gorman Tuura, Marianne Schmid Daners, Henning Richter

**Affiliations:** 1 Vascular Surgery, Hirslanden, 9016 St. Gallen, Switzerland; 2 Clinic for Cardiac Surgery, University Hospital Zurich, University of Zurich, 8091 Zurich, Switzerland; 3 Institute for Dynamic Systems and Control, Department of Mechanical and Process Engineering, ETH Zurich, 8092 Zurich, Switzerlandmarischm@ethz.ch (M.S.D.); 4 Department of Anesthesiology, Vetsuisse Faculty, University of Zurich, 8057 Zurich, Switzerland; 5 Diagnostic Imaging Research Unit (DIRU), Vetsuisse Faculty, University of Zurich, 8057 Zurich, Switzerland; fdelchicca@vetclinics.uzh.ch (F.D.C.);; 6 Surgery for Congenital Heart Disease, University Heart and Vascular Center Hamburg, 20251 Hamburg, Germany; 7 Center for MR Research, Children’s Hospital, University of Zurich, 8008 Zurich, Switzerland

**Keywords:** 3R, large animal models, cardiovascular research, translational research, pig models, disease models

## Abstract

Background and Purpose: Neurological injury remains a major complication of pediatric cardiac surgery and is closely related to alterations in cerebral blood flow during extracorporeal circulation (ECC). However, the real-time assessment of cerebral perfusion under these conditions has been limited by the lack of magnetic resonance (MR)-compatible perfusion systems. The aim of this pilot feasibility study was to establish a porcine model enabling simultaneous cardiopulmonary bypass (CPB) and real-time MR-based assessment of cerebral blood flow during simulated pediatric cardiac surgery. Methods: We conducted a pilot study on 11 Duroc pigs (14.6 ± 1.4 kg BW), designed in iterative cycles. The experimental setup included an MR-conditional heart-lung machine and a surgical protocol closely mimicking pediatric cardiac surgery. After the initiation of CPB and hemodynamic stabilization, animals were cooled to target temperatures (20 °C or 28 °C) depending on the perfusion strategy. Structural and functional MRI, including phase-contrast imaging, arterial spin labeling, diffusion-weighted imaging, and MR spectroscopy, were performed during cooling and rewarming. Procedural feasibility, technical challenges, and optimization strategies were systematically documented. Results: The study successfully established a reproducible porcine model enabling MR imaging during extracorporeal circulation. Key technical challenges, including vascular access, cannulation of the ascending aorta, and blood volume management, were identified and addressed through the iterative refinement of the surgical and perfusion protocols. The use of the Seldinger technique significantly improved cannulation safety and reduced blood loss. Stable CPB conditions and target hypothermic temperatures were achieved in successfully cannulated animals. MRI acquisition during CPB was feasible, providing simultaneous structural and functional assessment of cerebral perfusion. Representative imaging data demonstrate the capability of the model to capture cerebral hemodynamics in real time. Conclusions: This pilot study establishes a novel MR-compatible porcine model for the real-time assessment of cerebral blood flow during extracorporeal circulation. The platform provides a robust foundation for future quantitative investigations of cerebral perfusion, mechanisms of brain injury, and neuroprotective strategies in pediatric cardiac surgery.

## 1. Introduction

Animal models are essential for understanding disease mechanisms [1,2,3]. Cerebral perfusion during extracorporeal circulation (ECC) is complex. It is influenced by many physiological and technical variables [4,5,6]. Neurological impairment is one of the most serious complications of pediatric cardiac surgery. Common neurological lesions include ischemic stroke and cerebral hemorrhage. White matter injury, characterized by axonal damage and myelin disruption, is another clinically relevant brain injury [7]. These white matter lesions (WMLs) mainly result from reduced cerebral perfusion. In children with congenital heart defects (CHD), both the cardiac anatomy and immature cerebral autoregulation of blood flow influence it [8]. The link between WMLs and neurodevelopmental outcome has been well documented in pediatric cardiac surgery [9,10,11,12,13,14,15]. Studies in this field have become increasingly standardized [16,17].

Non-invasive methods, like near-infrared spectroscopy (NIRS), can be used to monitor cerebral blood flow, regional cerebral oxygen saturation (rScO2), and hemodynamic stability [18,19].

These methods have significant limitations compared to magnetic resonance (MR) technology. NIRS is not quantitative and cannot measure absolute cerebral blood perfusion or oxygenation. It also has poor reproducibility and inter-subject reliability [20].

Deep hypothermic circulatory arrest in infants with aortic arch obstruction seems to increase the rate of white matter injury [21]. These white matter lesions are associated with gross motor dysfunction [22,23,24]. Some lesions found by MR imaging (MRI) are initially silent. Adverse effects may only appear later [11,22,25]. The negative effects of CHD and surgery continue into adulthood and can impact children’s social environment [26]. Despite extensive clinical and neuroimaging data, basic questions remain about the timing and mechanisms of brain injury. Intraoperative factors also need exploration. MRI studies have revealed pre- and postoperative cerebral changes [27,28]. MRI is useful to analyze the extent and spatial distribution of perfusion during surgery. Its main advantage is its sensitivity to early ischemic changes when used with perfusion imaging. This helps identify vulnerable brain regions and determine injury timing, depending on heart-lung machine (HLM) settings and cooling protocols. MRI is therefore the preferred method for assessing white matter integrity, including WML detection [29,30]. However, real-time measurement of the effects of cardiopulmonary bypass (CPB) on cerebral perfusion has been difficult due to the lack of MR-conditional perfusion equipment.

With an MR-conditional HLM, we aim to fill this gap. After developing an initial prototype [31] and refining it [32,33], we plan to investigate the influence of CBP on cerebral perfusion. The objective of this study is to establish a pig model for simultaneous CPB and real-time MRI-based assessment of cerebral perfusion during simulated pediatric cardiac surgery. The pig model will serve as a platform for future mechanistic studies of brain vulnerability, perfusion strategies, and neuroprotective interventions.

## 2. Method

This animal study was approved by the cantonal veterinary authorities (license number ZH161/2022) and conducted in accordance with the Directive 2010/63/EU on the protection of animals used for scientific purposes and the Guide for the Care and Use of Laboratory Animals.

### 2.1. Study Rationale

The objective of this pilot study was to establish an animal model in pigs that enables simultaneous assessment of CPB performance and cerebral blood flow dynamics during experimental cardiac surgery.

This work serves as a platform for translational research on perfusion strategies, including cannulation techniques, flow and pressure regulation, and temperature management, with the overarching goal of identifying risk factors for cerebral injury and optimizing settings of the MR conditional HLM.

### 2.2. Methodological Approach

**Figure 1:**
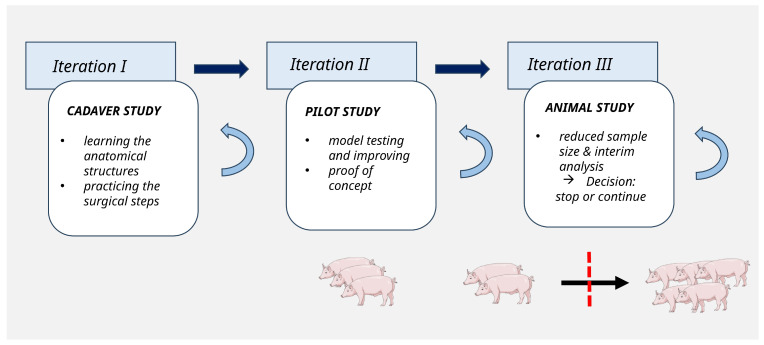
Illustration of the iterative refinement strategy using a structured, multistage development framework.

A robust and ethical large-animal model requires iterative refinement, especially when multiple procedural variables need to be optimized. We used a structured, multistage development framework for this pilot study (Figure 1). Initially, all surgical steps were practiced on cadavers to gain anatomical familiarity and test feasibility. Each step was evaluated, and contingency strategies were defined before the next iteration.

**Figure 2:**
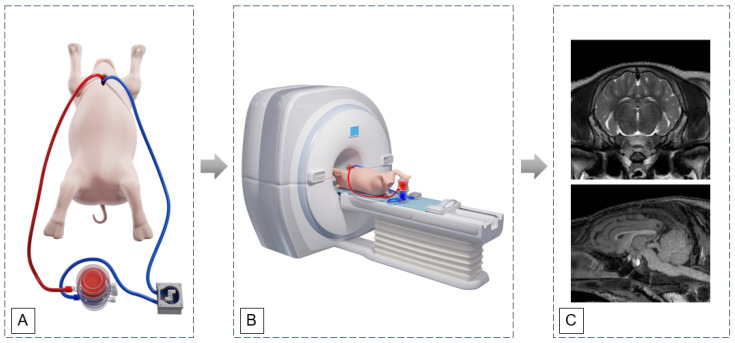
Illustration of the workflow of the CPB installation with the MR-conditional pump outside the MR room (**A**), MR imaging inside the scanner room (**B**), and representative transverse T2-weighted (**top**) and sagittal T1-weighted (**bottom**) images of the pig brain (**C**).

Protocols for anesthesia, surgery, imaging, and perfusion management were developed to minimize error sources and improve reproducibility. This highly interdisciplinary approach requires a wide range of experts, comprising veterinarians, clinicians, anesthetists, perfusionists, biomedical and imaging scientists. The number of animals was intentionally minimized in accordance with the 3R principles, and interim analyses were used to determine the necessity of protocol adjustments or the termination of specific study branches. Based on a successful proof-of-concept using the animal model, procedural feasibility could be shown. Following the pilot study establishing the pig model (Figure 2A) that allows assessment of CPB performance and cerebral blood flow dynamics under conditions similar to cardiac surgery, future studies will profit of using it as a platform for complex experiments about of brain vulnerability, perfusion strategies, and neuroprotective interventions [34].

### 2.3. Study Population

Eleven juvenile duroc pigs (5 male, 6 female) with an average weight of 14.6 kg (13.2–17.0 kg) and an average age of 54.2 days (42–68 days) underwent an open-heart surgery under general anesthesia.

After connection to the HLM and brief hemodynamic stabilization, each animal was transferred to the MR room under cardiopulmonary support and positioned in dorsal recumbency with the head oriented towards the gantry (Figure 2).

### 2.4. Study Design

**Figure 3:**
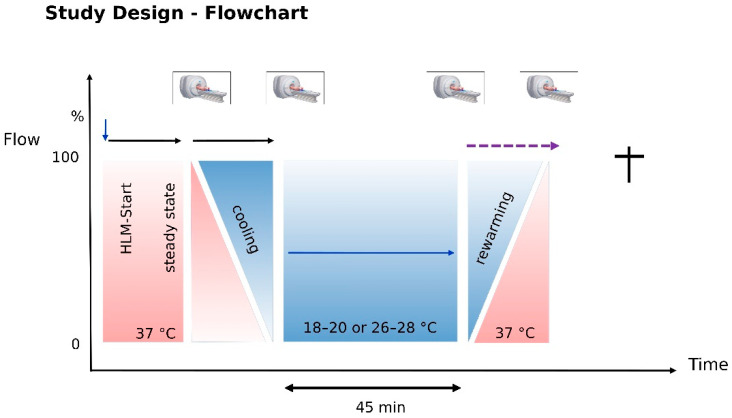
Study sequence and the timing of the MR acquisition (100% flow corresponds to 67 mL kg^−1^) [35].

The pilot study design (Figure 3) simulates pediatric cardiac surgery. After CPB was started and stabilized, the animals were cooled to the target temperature, which varied depending on the perfusion strategy (20 °C for deep hypothermic cardiac arrest and 28 °C for unilateral or bilateral perfusion). After rewarming, the animals were euthanized, and the brain was sampled for histological examination. Structural and functional MR sequences were performed during cooling and rewarming, following the imaging protocol [34].

### 2.5. Anesthesia

General anesthesia was induced via a tight-fitting face mask using a prefilled circle system delivering 7% sevoflurane in 100% oxygen through an advanced breathing system at a flow rate of 4 L/min. Spontaneous respiration was continuously monitored by capnography and end-tidal carbon dioxide (ETCO_2_) measurements. During mask induction, end-tidal sevoflurane concentrations were 5.00 ± 0.50%. Intravenous access was subsequently established via the caudal auricular veins for medication administration, and an arterial catheter was inserted into the femoral artery for continuous intra-arterial blood pressure monitoring. Following tracheal intubation, general anesthesia was maintained with propofol (6 mg/kg/h), midazolam (1 mg/kg/h), ketamine (0.50 mg/kg/h), and remifentanil (0.10 µg/kg/min) to ensure an adequate depth of anesthesia and analgesia. Infusion rates were adjusted according to the depth of anesthesia (DoA). Assessment of DoA included the evaluation of motor responses to stimulation (nasal septum pinch, tail clamp, claw clamp, and responses to painful or surgical stimuli), muscle tone, anal tone, corneal reflex, heart rate, and arterial blood pressure. Pancuronium was administered as an initial bolus (0.05 µg/kg) followed by continuous infusion (0.25 mg/kg/h) to achieve neuromuscular blockade. During instrumentation, piglets were initially ventilated using synchronized intermittent mandatory ventilation in pressure-controlled mode prior to neuromuscular relaxation. Subsequently, pressure-controlled ventilation was continued until surgical requirements necessitated a transition to volume-controlled ventilation. Respiratory rate, peak inspiratory pressure, and tidal volume were adjusted to maintain appropriate ETCO_2_ values within normal physiological ranges. Mechanical ventilation was adjusted to maintain an arterial partial pressure of carbon dioxide between 35 and 45 mmHg and arterial oxygen saturation between 98% and 100%. Fluid management consisted of a 1% glucose-containing Ringer’s lactate solution administered at 10 mL/kg/h. During CPB, oxygenation was regulated while maintaining pH management according to the alpha-stat strategy, ensuring that blood gas values were interpreted relative to body temperature [36].

Intensive monitoring was performed throughout the whole anesthesia process, including paremeters such as electrocardiogram, heart rate, pulse rate and invasively measured blood pressures (systolic, mean and diastolic) via an arterial catheter in an auricular artery. Furthermore, inspired and expired concentrations of carbon dioxide, oxygen and isoflurane, as well as esophageal temperature and saturation of arterial blood, were monitored. All parameters were constantly measured and recorded in 10 min intervals. At the end of the experiment, animals were euthanized under deep anesthesia by the slow intravenous administration of T61 (0.3–0.5 mL/kg body weight; combination of embutramide, mebezonium iodide, and tetracaine hydrochloride; MSD Animal Health, Switzerland). Death was confirmed by the absence of vitral signs, cardiac activity and respiration.

### 2.6. Surgery

All surgical instruments were standard clinical-grade instruments obtained from established manufacturers (e.g., B. Braun/Aesculap, Germany; Fine Science Tools, Germany), which are routinely used in cardiovascular surgery (Table 1). Instrument selection followed the surgeon’s preference. Equivalent instruments from other certified manufacturers can be used interchangeably without affecting the procedure. After confirming a surgical plane of anesthesia, a standardized median sternotomy was performed.

#### 2.6.1. Installation of the Cardiopulmonary Bypass

The surgical field was disinfected and draped. Hemostasis of bony and periosteal structures was achieved using electrocautery and bone wax. The proximal sternum is relatively thick in young pigs, requiring careful dissection to avoid venous bleeding from the thymic and mediastinal tissues. Careful dissection is performed directly along the bone until the retrosternal tissue is free of tension. Over-dissection of the pericardial reflections should be avoided to minimize bleeding risk, including adventitial hemorrhage (treatment option: compression until bleeding stops).

#### 2.6.2. Cannulation

Extracorporeal circulation began with arterial cannulation, followed by venous cannulation. To ensure suitability in the magnetic field, it was necessary to replace commercially available cannulas with MR-conditional alternatives. Old Edwards cannula (Remnants, LifeStream 10 Fr) was used. The venous cannula was a left heart vent cannula (Medtronic, Art 12008), with the tip modified by cutting it off at a slight angle. An oblique cutting facilitated the cannulation of the vessel. The cannula’s various openings, located at the tip and on the sides, facilitate drainage and minimize suction-related issues.

#### 2.6.3. Arterial Cannulation

**Figure 4:**
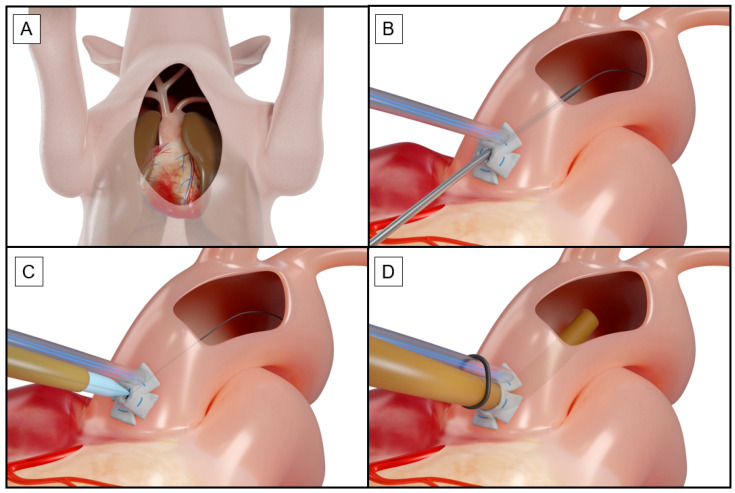
Arterial cannulation using the Seldinger technique/anatomy after sternotomy (**A**), puncture and insertion of a wire inside the aortic arch (**B**), introduction of the aortic canula (**C**), and position of the canula in the aortic arch (**D**).

Following median sternotomy (Figure 4A), two purse-string sutures (5-0 or 6-0 Prolene, Ethicon, part of the Johnson & Johnson Medical Devices Companies) were placed with soft Teflon pledgets (Figure 4B). The Seldinger technique was then used to puncture the vessel wall and insert a soft wire (Figure 4B,C: Rosen wire and a hydrophilic J-tip wire (Terumo)). This step was always performed by two surgeons: one to perform the procedure and the other to fix the wire. When inserting the arterial cannula, the dilator of a 5Fr sheath was used to stabilize the Edwards cannula (Figure 4C,D).

#### 2.6.4. Venous Cannulation

The right atrium or the superior vena cava was equally suitable for venous cannulation. After placement of two purse-string sutures (heart ear: 4-0 or 5-0 Prolene; SVC: 5-0 Prolene), an additional suture was applied as needed to ensure hemostasis. Venous cannulation was also performed using the Seldinger technique, which obviated the need for surgical incision of the right atrium or superior vena cava and reduced potential blood loss. An introducer was recommended during venous cannula insertion to prevent retrograde blood flow into the cannula and leakage through lateral perforations. Following successful cannulation and achievement of the target activated clotting time (ACT), the HLM was connected.

#### 2.6.5. Connection to the HLM

During the initial phase of extracorporeal circulation, a conventional electrically driven HLM (LivaNova S5, London, UK) was used. This system allowed the initiation of the CBP at lower rotational speeds and facilitated retrograde priming of the circuit with blood to minimize hemodilution. After hemodynamic stabilization of the animal, perfusion was transitioned to the MR-conditional HLM, as previously described [32,33].

#### 2.6.6. MR-Conditional Heart-Lung Machine

The MR-conditional HLM comprises a pump unit with a modified roller pump head (SPQ 225; Möller Medical GmbH, Fulda, DE), an air motor (PM0450, PTM mechatronics GmbH and Bibus AG, Fehraltorf, CH), and an encoder (ME 22 LD; PWB encoders GmbH, Eisenach, DE) (32,33). The prototype design was based on the S3 SPQ 225 pump from Stöckert (Sorin Group, Milano, IT), which incorporated two rollers. Ferromagnetic components were replaced with custom-developed MR-conditional components. A Paragon Infant Oxygenator (Chalice Medical Europe GmbH, DE) was used. The oxygenator has a priming volume of 150 mL and supports blood flow rates of 0.5 to 3 L/min. The CPB flow was calculated as 67 mL/kg body weight (34). During MRI acquisition, the HLM was positioned directly adjacent to the animal or as close as possible to its caudal end (Figure 2B). At a circuit length of approximately 60 cm, the total priming volume, including the oxygenator and complete tubing system, was approximately 190–200 mL.

**Figure 5:**
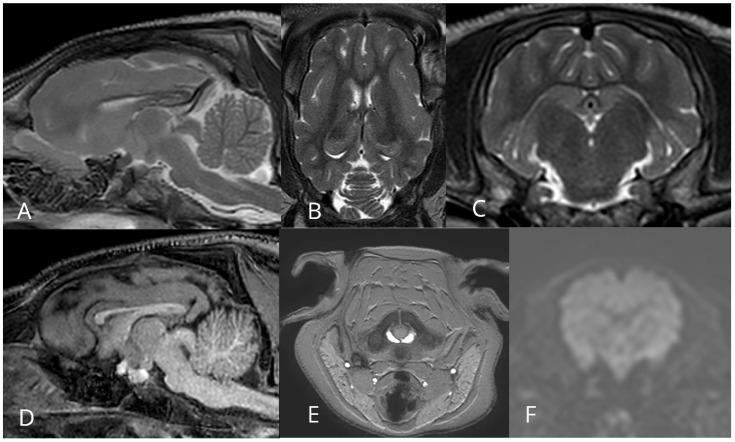
Representative magnetic resonance imaging (MRI) of a pilot animal. (**A**–**D**) Morphological MRI: T2-weighted images in sagittal (**A**), coronal (**B**), and axial (**C**) planes and a T1-weighted sagittal image (**D**). (**E**,**F**) Functional MRI: axial phase-contrast image with velocity encoding at the level of the first cervical vertebra for assessment of blood flow in the carotid arteries and jugular veins (**E**), and axial diffusion-weighted image (**F**) illustrating water molecule diffusion in the brain. Note the comparatively lower spatial resolution of the diffusion-weighted image.

**Figure 6:**
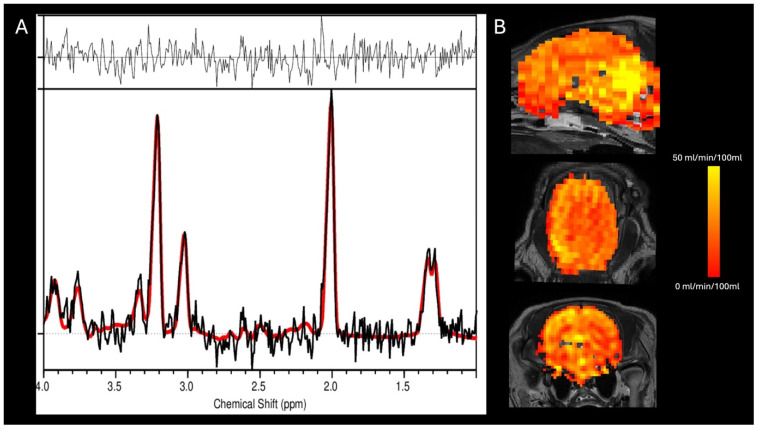
Representative magnetic resonance (MR) spectroscopy and arterial spin labeling (ASL) imaging from a pilot animal. (**A**) MR spectrum showing the acquired spectral data (black) and the corresponding spectral fit (red), along with the residuals. (**B**) Triplanar ASL perfusion maps overlaid on anatomical images, displayed in sagittal, coronal, and axial orientations. Color scale indicates cerebral blood flow (0–50 mL/min/100 mL).

After reconnection of all equipment and administration lines, imaging was performed using a 16-channel head coil according to a study-specific MRI protocol (Table 2). Brain morphology was examined using T2-weighted Turbo Spin Echo sequences acquired in multiple orientations, complemented by a sagittal 3D T1-weighted Turbo Field Echo sequence. Hemodynamic parameters in major cervical vessels were quantified with phase-contrast MRI, positioning the imaging plane orthogonal to the carotid arteries and jugular veins, with flow gating synchronized to pump-induced pulsations. Water diffusion properties in brain tissue were assessed via diffusion tensor imaging, including analysis of temperature dependence using linear fitting. Cerebral perfusion was measured with pCASL, and metabolic information was obtained using single-voxel PRESS spectroscopy (Figure 5 and Figure 6). This protocol enabled the assessment of cerebral perfusion and metabolic parameters under different HLM configurations. This work focuses on the surgical procedure; the detailed results of MRI investigations from subsequent experiments are reported separately [34].

## 3. Results

The proposed porcine model was successfully established, enabling simultaneous assessment of CPB performance and cerebral blood flow dynamics during experimental cardiac surgery.

Cadaver study: A detailed assessment of the porcine anatomy and the feasibility of the surgical approach was conducted using cadavers. Surgical steps were performed by two cardiac surgeons with 5 and 10 years of experience, respectively, both trained in pediatric cardiac surgery and in endovascular surgical techniques. Direct surgical cannulation was selected to ensure procedural consistency with pediatric cardiac surgery practice.

**Figure 7:**
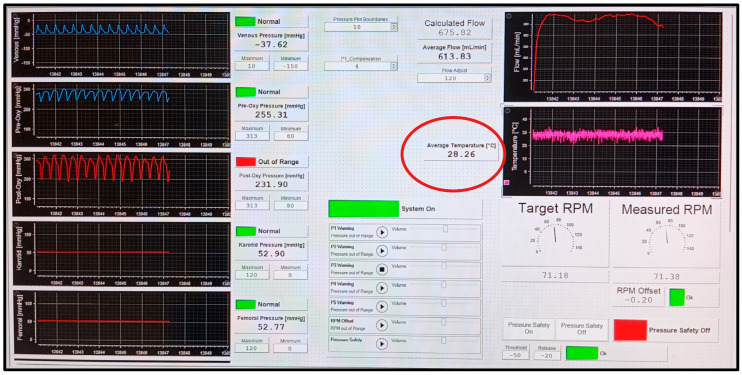
Screenshot shows exemplary online data recording—immediately before reaching the target temperature of 28 °C (red circle).

Pilot study: The baseline characteristics of the pilot study cohort are summarized in Table 3. In successfully cannulated animals, an expected decrease in hematocrit was observed within the first two hours of cardiopulmonary bypass. The median baseline hematocrit was 28% (range 25–32%). In some animals, the study was terminated prematurely due to severe pericardial adhesions associated with pericarditis (Animal No.2), resulting in major bleeding due to right atrial injury during cannulation (Animal No. 4), severe bleeding and vena cava collapse correlating with substantial blood loss (Animals No. 3, 8 and 11), and complications during intubation (Animal No. 6). Despite these initial challenges, the pilot study demonstrated the feasibility of performing the MRI protocol as planned. In the nine successfully cannulated animals, the target hypothermic temperatures of 28 °C and 20 °C were achieved (Figure 7).

Compared to pediatric cardiac surgery, porcine aortic tissue is more fragile. Bleeding during cannulation of a short ascending aorta, subadventitious bleeding, and accidental lacerations result in minimal but unacceptable blood loss for the planned study. It was therefore decided to use an alternative cannulation technique (Seldinger technique) for subsequent operations. The successful completion of the pilot study enabled the evaluation of different perfusion strategies, including cannulation approaches (unilateral vs. bilateral antegrade cerebral perfusion) and perfusion parameters (e.g., temperature, blood pressure, or blood flow) [34].

## 4. Discussion

This pilot study aimed to develop and establish a porcine MRI model that enables simultaneous assessment of the CPB performance and cerebral blood flow dynamics under conditions closely resembling pediatric cardiac surgery. The principal finding is the successful establishment of a complex experimental platform that integrates an MR-conditional HLM with real-time MRI, allowing investigation of cerebral perfusion during extracorporeal circulation. Importantly, this study focuses on the technical feasibility and procedural optimization of the model rather than on quantitative physiological analysis.

### 4.1. Avoidance of Blood Donation

The key methodological decision to distinguish this model from previously published small- and large-animal studies was the deliberate choice to avoid additional blood donors [4]. This choice is particularly challenging in juvenile pigs, as rapid postnatal growth is associated with relatively low baseline hemoglobin and hematocrit levels. Nevertheless, hematocrit values remained sufficiently stable for at least two hours following cannulation and connection to the MR-conditional HLM, demonstrating the feasibility of a blood-sparing strategy in this setting.

This outcome can be attributed to two main factors. First, the MR-conditional HLM can be positioned in close proximity to the animal, thereby minimizing tubing length and reducing circuit priming volume. In contrast, other experimental setups require extended tubing due to the incompatibility of conventional circulatory support devices with the MRI environment [5], resulting in substantially higher priming volumes and necessitating larger animals or blood supplementation [37]. Second, the choice of cannulation strategy was critical. Central cannulation was selected to closely replicate pediatric cardiac surgical practice and to ensure effective venous drainage during prolonged experimental protocols [38]. This approach has also been successfully applied by other groups working with animals of comparable age and size [4].

### 4.2. Cannulation Strategy and Surgical Considerations

During the pilot phase, different cannulation techniques were evaluated to optimize reproducibility and minimize blood loss. Two approaches were considered particularly useful: direct cannulation following partial unclamping with a Satinsky clamp under optimal exposure, and cannulation using the Seldinger technique in cases of limited exposure, such as a short ascending aorta, cardiac rotation, or overlapping anatomical structures. Owing to its simplicity, reproducibility, and minimal blood loss, the Seldinger technique was ultimately favored and adopted for subsequent procedures. This decision was further supported by the observation that porcine aortic tissue is more fragile than that typically encountered in pediatric cardiac surgery. Cannulation of a short ascending aorta was associated with subadventitial bleeding and occasional accidental lacerations, resulting in minimal yet unacceptable blood loss for a blood-sparing study design. Transitioning to the Seldinger technique significantly reduced these risks and improved procedural safety.

Delayed administration of full systemic heparinization until completion of surgical preparation and arterial cannulation was also found to be advantageous. During the brief interval following cannulation, the risk of thrombus formation in the high-flow, high-pressure arterial system was considered low. Alternatively, venous cannulation with gradual drainage initiation can be performed first to reduce arterial pressure before arterial cannulation, although this approach entails arterial cannulation under anticoagulation.

To further minimize bleeding complications in anticoagulated animals, meticulous surgical technique is essential, especially given the severely restricted access to the animal in the MR scanner. Restrained tissue dissection, immediate control of bleeding using electrocoagulation or hemostatic agents, and ligation of small vessels when necessary are critical measures. The use of vacuum-assisted blood retransfusion systems, or preferably a cell saver, is strongly recommended as long as possible before entering the MR scanner, particularly in the absence of blood donors. Closure of the sternum or skin to achieve tissue compression, insertion of a chest drain to detect early intrathoracic bleeding, and continuous monitoring of blood loss further contribute to procedural safety.

The integration of MRI into the experimental setup represents a central innovation of this model. Structural and functional MRI acquisitions, including phase-contrast imaging, arterial spin labeling, diffusion-weighted imaging, and MR spectroscopy, were successfully performed during extracorporeal circulation. Representative imaging data demonstrate the capability of the model to capture cerebral perfusion and metabolic information in real time. However, quantitative analysis of cerebral blood flow and related parameters was beyond the scope of this pilot feasibility study and is reported separately [34]. The present work therefore provides the methodological foundation for such analyses.

### 4.3. Relevance for Cerebral Injury and Perfusion Research

Surgical procedures involving the aortic arch remain associated with substantial morbidity and mortality [39,40,41]. One of the central motivations of this model is to investigate the timing, distribution, and mechanisms of cerebral injury, such as white matter lesions, that are associated with neurodevelopmental deficits following pediatric cardiac surgery [9,10,11,12,13,14]. In contrast to previous approaches relying on microsphere injection or post-mortem histology, which do not allow dynamic or longitudinal assessment, the present model enables real-time, noninvasive monitoring of cerebral perfusion under controlled experimental conditions [42,43,44,45,46].

The established platform enables the systematic investigation of clinically relevant variables, including cannulation strategies (e.g., unilateral versus bilateral antegrade cerebral perfusion), temperature management, perfusion pressure, and flow conditions [34]. In addition, the model provides a framework for evaluating pharmacological neuroprotective interventions and for integrating experimental data into predictive computational models of cerebral hemodynamics.

### 4.4. Rationale for a Porcine Model

A translational large animal approach is essential for investigating pathophysiological mechanisms relevant to pediatric cardiac surgery. Pigs are widely regarded as a suitable model for cardiovascular research due to similarities to humans in cardiac anatomy, physiology, and metabolism [47,48,49,50,51]. The porcine brain also shares important developmental and structural characteristics with the human brain, particularly in the perinatal period. These similarities include patterns of brain growth, myelination, and susceptibility to hypoxia-related injury, making the pig an appropriate model for studying cerebral vulnerability in the context of congenital heart disease [21,52,53,54,55,56].

To balance the requirements of circulatory support with blood conservation, catheter length and circuit volume were minimized by positioning the HLM as close to the animal as possible. In addition, an iterative refinement strategy aligned with the 3R principles was implemented, allowing continuous optimization of the experimental protocol while minimizing animal use [57,58]. Standardized procedures for anesthesia, surgery, and MRI acquisition further enhance reproducibility and facilitate future application of the model.

### 4.5. Limitations

This study has several limitations. The number of animals was limited, reflecting the pilot and feasibility-driven nature of the study. Consequently, the study was not designed or powered for statistical analysis or hypothesis testing. Second, MR-conditional cannulas used in this study were not originally designed for this specific application, requiring compromises between MR compatibility and optimal mechanical performance. Third, blood re-transfusion systems could only be used prior to positioning the animal in the MR scanner, resulting in limited control of blood loss during imaging. Finally, prolonged experimental duration increases the risk of complications, including bleeding under anticoagulation and intravascular volume loss associated with systemic inflammatory responses. These factors highlight the importance of continued refinement of the protocol.

## 5. Conclusions

In conclusion, this pilot study demonstrates the successful establishment of an MR-compatible porcine model that allows the integration of extracorporeal circulation with real-time MRI. The developed platform enables simultaneous assessment of cerebral perfusion and structural brain parameters under conditions approximating pediatric cardiac surgery.

By combining an MR-conditional HLM with refined surgical and perfusion techniques, this model provides a robust and reproducible experimental framework. While the present study focuses on methodological development rather than quantitative physiological analysis, it establishes the technical foundation for future investigations of cerebral blood flow dynamics, mechanisms of brain injury, and neuroprotective strategies during cardiopulmonary bypass.

## Figures and Tables

**Table 1:** Surgical equipment (minimum configuration).

Category	Instrument/Material	Manufacturer Details
Scissors	Stevens/Iris scissors	B. Braun Melsungen AG (Germany); Fine Science Tools (Germany)
	Metzenbaum scissors	Aesculap (B. Braun), Germany
	Mayo scissors	KLS Martin Group, Germany
Forceps	Adson/DeBakey forceps	B. Braun Melsungen AG, Germany
	Micro-tweezers	Fine Science Tools, Germany
Clamps	Backhaus cloth clamp	Aesculap (B. Braun), Germany
	Halstead-Mosquito clamp	KLS Martin Group, Germany
	Rochester Pean clamp	B. Braun Melsungen AG
	Tube clamp	Aesculap (B. Braun)
Scalpel	Blade holder + blades No. 10, 11	Swann-Morton, UK
Needle Holder	Castro-Viejo micro needle holder	Fine Science Tools
	Neivert needle holder	Aesculap (B. Braun)
Introducer Set	Puncture cannula	Terumo Corporation, Tokyo, Japan
	Guidewire (Rosen/hydrophilic J-tip)	Terumo Corporation
	Dilators (5 Fr, 12 Fr)	Medtronic, USA
Arterial cannula	Remnants, LifeStream 10 Fr	Edwards Lifesciences, USA
Venous cannula	Left heart vent cannula, Art 12008	Medtronic, USA
Suture Material	Prolene 3-0, 4-0, 5-0	Ethicon (Johnson & Johnson)
	Teflon pledgets	W. L. Gore & Associates
Additional Material	Vacuum blood reservoir	Getinge
	Cell Saver	LivaNova PLC
	Oscillating saw	Stryker Corporation
	Electrocautery	Erbe Elektromedizin
	Defibrillator pads	Philips
	Vessel loops	Medline Industries
	Surgical drapes, gauze	Paul Hartmann AG
	PPE & disinfectant	3M, Switzerland

**Table 2:** Overview of magnetic resonance imaging (MRI) protocol.

Scan Name	Description	TR/TE (ms)	FoV (mm^2^)	AM	RM	ST (mm)	Acquired Data
T2W_TSE	T2-weighted turbo spin echo	2000–7993/80–100	a.t.a	168–236 × 104–155	288 × 288 to 528 × 528	1.5	T2: cerebrospinal fluid, fluid-filled ventricles and fluid within the parenchyma
sT1W_3D	T1-weighted 3D view	13./5.9	a.t.a	168 × 167	240 × 240	0.6	T1: morphology of the brain
TOF	time-of-flight angiography	23/3.45	200 × 200 × 102	200 × 166	Reconstruction Voxel 0.39 × 0.39 × 0.6		vascular anatomy, and qualitative assessment of vascular flow
PC_venc	phase contrast with velocity coding	11/7.1	150	256 × 179	Reconstruction Voxel 0.59 × 0.59 × 4.0	4	quantitative assessment of blood flow
3d_pCASL	pseudo-continuous arterial spin labeling	4171/13	170 × 170	96 × 96	64 × 60	4	quantitative perfusion (blood flow/volume)
DTI	diffusion tensor imaging	3822/91	a.t.a	194 × 102	128 × 128	2	diffusion and direction process of water and other molecules in tissue
SV_PRESS	single voxel point resolved spectroscopy	2000/288	(Volume of interest: 10 × 10 × 27.54)	-	-	-	chemical composition of tissue at a single ROI (region of interest)
2D_PRESS	multi voxel point resolved spectroscopy	2000/144	230 ×192	23 × 19	REC Voxel 9.58 × 9.58 × 15.0	15	chemical composition of tissue in multiple ROIs

**Table 3:** Overview of the pilot study including remarks (m: male; f: female; BW: body weight). The main foci of this work are animals 2 and 4. Animals 7–10 are presented in detail in [34].

No.	Sex/Weight	Cardiac Output (67 mL/kg BW)	Hematocrit (%—pre/Post Cannulation)				Target Temperature (°C—r = Rectal, t = Thoracic)	Remarks
	**(m/f)/(kg)**		Start	Post Surgery	1 h	2 h		
1	m/16	1072.0	26	23	21	19	28 (r)	
2	m/13.9	931.3	30	-	-	-	28 (r)	pronounced pericardial adhesions (pericarditis), Hematocrit missing
3	m/13.2	884.4	29	25	24	23	28 (r)	Collapse of vena cava
4	m/14.8	991.6	25	-	-	-	28 (r)	atrial tear and massive blood loss
5	f/16.5	1105.0	30	31	34	20	20 (r)	
6	f/17.0	1139.0	-	-	-	-	28 (r)	Complications during intubation
7	m/14.0	938.0	25	25	23	22	28 (r)	
8	f/15.0	1005.0	28	26	23	22	28 (r)	Collapse of vena cava
9	f/13.4	897.8	30	25	24	21	28 (r)	
10	f/14.0	938.0	32	31	21	24	28 (t)	
11	f/13.0	871.0	27	18	11	7	20 (t)	Substantial bleeding

## Data Availability

The original contributions presented in this study are included in the article. Further inquiries can be directed to the corresponding author.
